# Proprioceptive Training Improves Postural Stability and Reduces Pain in Cervicogenic Headache Patients: A Randomized Clinical Trial

**DOI:** 10.3390/jcm13226777

**Published:** 2024-11-11

**Authors:** Mohamed Abdelaziz Emam, Tibor Hortobágyi, András Attila Horváth, Salma Ragab, Magda Ramadan

**Affiliations:** 1 Basic Sciences Department, Faculty of Physical Therapy, Kafrelsheikh University, Kafr El-Sheikh 33511, Egypt; 2 János Szentágothai Neurosciences Division, Semmelweis University, 1085 Budapest, Hungary; 3 Department of Kinesiology, Hungarian University of Sports Science, 1123 Budapest, Hungary; t.hortobagyi@umcg.nl; 4 Department of Sport Biology, Institute of Sport Sciences and Physical Education, University of Pécs, 7622 Pécs, Hungary; 5 Center for Human Movement Sciences, University of Groningen Medical Center, University of Groningen, 9713 AV Groningen, The Netherlands; 6 Neurocognitive Research Centre, Nyírő Gyula National Institute of Psychiatry and Addictology, 1135 Budapest, Hungary; 7 Department of Anatomy Histology and Embryology, Semmelweis University, 1094 Budapest, Hungary; 8 Research Centre for Natural Sciences, Hungarian Research Network, 1117 Budapest, Hungary; 9 Department of Neuropsychiatry, Faculty of Medicine, Kafrelsheikh University, Kafr El-Sheikh 33511, Egypt; 10 Basic Sciences Department, Faculty of Physical Therapy, Cairo University, Giza 12613, Egypt; magdaramadan2000@yahoo.com

**Keywords:** gaze direction recognition, balance, motor imagery, neck pain, HUMAC balance system

## Abstract

**Background:** Headache is one of the leading causes of disability in the world. Neck proprioception, pain, and postural control are interconnected in both healthy individuals and those with chronic neck pain. This study examines the effects of proprioceptive training using a gaze direction recognition task on postural stability and pain in cervicogenic headache patients. **Methods:** Patients with cervicogenic headache (*n* = 34, age: 35–49 y) were randomized into a control group (CON), receiving only selected physical therapy rehabilitation or to an experimental group (EXP), performing proprioceptive training using a gaze direction recognition task plus selected physical therapy rehabilitation. Both programs consisted of 24, 60 min long sessions over 8 weeks. Postural stability was assessed by the modified clinical test of sensory integration of balance (mCTSIB) and a center of pressure test (COP) using the HUMAC balance system. Neck pain was assessed by a visual analog scale. **Results:** In all six tests, there was a time main effect (*p* < 0.001). In three of the six tests, there were group by time interactions so that EXP vs. CON improved more in postural stability measured while standing on foam with eyes closed normalized to population norms, COP velocity, and headache (all *p* ≤ 0.006). There was an association between the percent changes in standing on foam with eyes closed normalized to population norms and percent changes in COP velocity (r = 0.48, *p* = 0.004, *n* = 34) and between percent changes in COP velocity and percent changes in headache (r = 0.44, *p* = 0.008, *n* = 34). **Conclusions:** While we did not examine the underlying mechanisms, proprioceptive training in the form of a gaze direction recognition task can improve selected measures of postural stability, standing balance, and pain in cervicogenic headache patients.

## 1. Introduction

Headache is one of the leading causes of disability in the world [1]. Cervicogenic headache (CGH) is a type of headache triggered by neck movements or pressure on tender points in the neck. CGH emanates from abnormalities in the cervical spine’s bony elements, intervertebral discs, and/or soft tissue elements, and CGH is often accompanied by neck pain as well [2,3]. These musculoskeletal dysfunctions may include movement restrictions in the upper cervical segments [4].

People who report CGH also suffer from comorbidities, including vestibular abnormalities and poor balance [5,6]. Impaired postural stability in CGH can arise through peripheral mechanisms. One suggestion is that neck proprioceptive afferents become impaired, affecting central and reflex connections between mechanoreceptors, visual, and vestibular systems [7,8]. Postural control needed for standing and walking stability, relies on sensory input from the vestibular, visual, and somatosensory systems. Proprioceptive feedback also aids postural control and arises from areas rich in such receptors in the upper cervical [9,10].

The upper cervical region, rich in proprioceptive receptors, informs the central nervous system about neck position [7]. Impaired cervical proprioception is thus central to sensorimotor control issues in neck pain patients [11]. Neck pain or neck muscle fatigue can distort sensory input from the neck proprioceptors and affect postural control [8,12]. The pathway by which pain originating in the neck can be referred to the head is the trigeminocervical nucleus, which descends in the spinal cord to the level of C3/4 and is in anatomical and functional continuity with the dorsal gray columns of these spinal segments [13]. The trigeminocervical nucleus is a region of the upper cervical spinal cord where sensory nerve fibers in the descending tract of the trigeminal nerve (trigeminal nucleus caudalis) are believed to interact with sensory fibers from the upper cervical roots. This functional convergence of upper cervical and trigeminal sensory pathways allows the bidirectional referral of painful sensations between the neck and trigeminal sensory receptive fields of the face and head [14].

Neck proprioception, pain, and postural control are interconnected in both healthy individuals and those with chronic neck pain [15]. Exercises that focus on sensorimotor function, particularly retraining cervical proprioception and neck muscle coordination, can improve postural control and alleviate CGH [16]. Integrating task-oriented training with neck movement control training has also been shown to improve neck pain and sensory abilities [17]. Thus, incorporating balance training through cervical region proprioceptive exercises is essential for addressing these sensorimotor problems.

Control of gaze direction is the result of coordinated eye and neck movements. The concept of sensory-motor conflict suggests that prolonged neck pain may result from an incongruence between sensory and motor feedback in the neck, compounded by visual-motor conflict in cortical areas associated with gaze direction [18]. Proprioceptive exercises and graded motor imagery, which enhance proprioceptive acuity and reduce joint position errors, are part of the training regimen. Graded motor imagery activates the higher-order motor cortex, while mirror therapy engages the primary sensory-motor cortex, improving visual-motor feedback and facilitating actual motor activity [19]. Clinical evidence supports the effectiveness of mirror therapy, especially in chronic pain cases.

Moreover, scientific progress in rehabilitation medicine shows that customizing the rehabilitation process by using functionally oriented movements, also known as task-specific training, may be beneficial for patients suffering from CGH. Along with the enhancement of proprioception, task-oriented exercises initiate neuroplastic changes in the brain, which facilitate restoration of sensorimotor functions [20]. This method integrates both the purpose of carrying out the proprioceptive exercises and the purpose of carrying out task-centered training. This method may be useful in addressing both the underlying cause and the symptoms of CGH.

However, there is a notable gap in direct experimental evidence showing that proprioception training can improve postural stability and reduce pain in CGH patients. To address this gap, we conducted a study using a mental motor imagery task known as gaze direction recognition (GDR). During GDR, patients observe the neck rotation of another individual from behind and attempt to recognize the direction of gaze. This process involves motor imagery, and previous studies have shown that GDR tasks significantly increase oxyHb concentrations in the premotor area and superior temporal sulcus compared to simple action observation [18]. The purpose of the present study was to examine, for the first time, the effects of a specific form of neck proprioception training—gaze direction recognition exercise (GDRE)—on postural stability and pain in patients with CGH. Based on the above outlined neurophysiological and neuroanatomical evidence, we hypothesized that GDRE compared with standard care will be more effective in improving measures of postural control and headache.

## 2. Materials and Methods

### 2.1. Design and Patients

**Figure 1:**
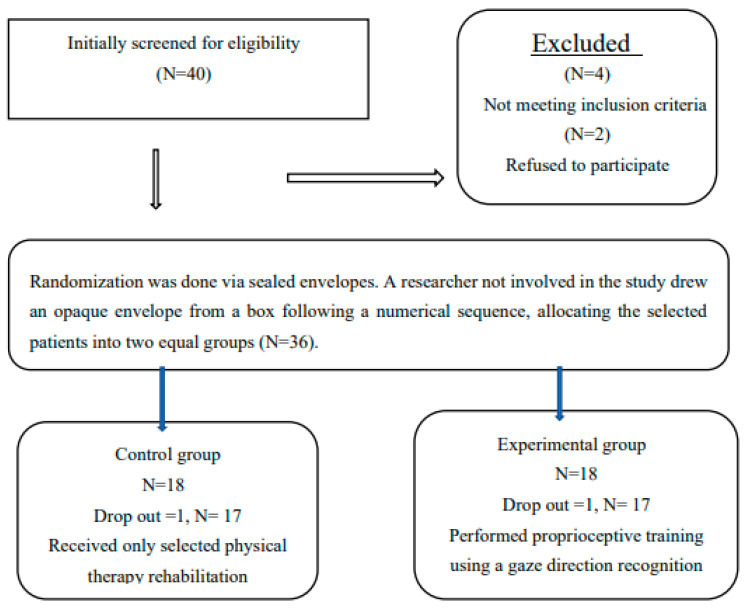
Flowchart of patient recruitment and study participation. This flowchart illustrates the number of patients screened, enrolled, and excluded at each stage of the study. It details the progression from initial recruitment through to final analysis, highlighting reasons for exclusion and the final sample sizes for the control (CON) and experimental (EXP) groups.

The study is a randomized clinical trial, conducted between December 2021 and November 2023. Each patient received detailed information about the protocol and signed an informed consent before the start of the measurements. The Cairo University Ethical Committee approved the study protocol (P.T.REC/012/004976), which was registered in the Pan African Registration Trials (PACTR202201829248437). Figure 1 shows the study design.

A priori sample size estimation was based on the hypothesized group by time interaction in G*Power. As reported in previous publications [16], the mean ± SD of cervical rotation ROM in study group was approximately 56.5 ± 11.3, while in control group it was approximately 46.7 ± 8.0. Using an effect size (f) of 0.25 and a power of 80%, a sample size of 17 patients in each group was needed to reject the null hypothesis. Assuming a 20% loss to follow-up, at least 20 patients were needed for each group.

Patients (n = 40) were recruited from Kafr Elsheikh University Hospital and Kafr Elsheikh General Hospital, Egypt. Inclusion criteria: physician-diagnosed CGH according to the current diagnostic criteria for CGH [2]; age 35 to 49 years; unilateral pain starting in the neck and radiating to the frontotemporal region; pain aggravated by neck movements; restricted cervical range of motion; joint tenderness in at least one of the joints of the upper cervical spine (C1–C3); and headache frequency of at least 1 per month continuously over the past year. Exclusion criteria: a history of head and neck injury and surgery; musculoskeletal problems/disorders; neurological problems/diseases; metabolic syndromes; hyper- or hypotension; vestibular disorders; and inner ear inflammation.

### 2.2. Interventions

Of the 40 patients recruited, six failed to complete the rehabilitation program. As outpatients, participants completed 24, 60 min long sessions over two consecutive months (i.e., 3 sessions/week). The control group (CON, n = 17) performed selected physical therapy rehabilitation exercises, consisting of 20 min of hot pack, 20 min of transcutaneous electrical nerve stimulation of the cervical area, and 5 min of ultrasound application to the neck and performed range of motion, postural, and isometric exercises (chin tuck) [19]. The experimental group (EXP, n = 17) received the selected physical therapy rehabilitation and additionally performed the gaze direction recognition exercise (GDRE) program for 10 min in each session. GDRE is a new practice used to improve the proprioception of cervical muscles and to rehabilitate patients with neck disability [16,19].

Briefly, during GDRE, the therapist sat in a chair 0.75 m behind the center of a wooden table (1.8 × 0.4 × 0.76 m). On the table edge near the therapist, six wooden blocks were placed 0.31 m apart and numbered 1 to 6 from left to right. Patients sat in a chair behind the therapist but were able to see the numbered wooden blocks. The therapist directed his gaze and head to one of the blocks at random. An assistant therapist signaled to the therapist to start the protocol and direct his visual attention to one of the blocks. As patients observed the experimenter’s neck rotation from behind, they followed the direction of rotation with their own head. Patients were asked to guess the block number the therapist was looking at and call out the number of this block as quickly as possible [16]. Patients received no feedback if they in/accurately guessed the block. The assistant therapist recorded the reaction time and whether the patient guessed the block number correctly or not. A single experimental GDR task consisted of 30 trials (10 min). Patients were instructed to rotate the head without moving other body parts [16].

### 2.3. Outcome Measures

Postural stability measured by the modified clinical test of sensory integration of balance (mCTSIB): The HUMAC balance system is reliable and valid to assess postural stability [20]. The system consists of a force platform (L: 0.5 m, W: 0.5, H: 0.05 m) with a grid to drawn on its surface to allow reproducible foot position and placement. A menu guides the therapist through the protocol of mCTSIB and the standing balance: The balance test measures the sensory integration of postural stability while standing on firm and foam surfaces with eyes open or closed (HSEO = hard surface eyes open, HSEC = hard surface eyes closed, SSEO = soft surface eyes open, SSEC = soft surface eyes closed). After positioning the feet on the platform grid, patients were asked to focus on the target on the monitor in the eyes-open condition and minimize sway without any visual feedback on the screen, as the target disappears after the test starts, for 30 s, a duration long enough to obtain sufficient data for analysis [21]. Patients were then instructed to repeat the test with eyes closed.

Patients repeated the eyes open and closed trials one more time, but this time while standing on a foam surface. Each condition was repeated three times and the best value of stability score (%) was used in the statistical analysis [22]. The rationale for using four different standing balance tests lies in the multifaceted nature of balance control, which involves the proprioceptive, visual, and vestibular systems. Each test is designed to isolate and challenge these constituents of standing balance. Because our proprioceptive training was designed to primarily improve the sense of body position but less so vision, we expected to find the greatest changes measured in the foam conditions [23].

In this test, we computed a stability score based on the center of pressure (COP) movement to a predefined area or range that represents stability. Stability score (%) = 100 × (1 − (area outside stability zone/total area of stability zone) * area outside stability zone = (stability zone area − COP excursion area). Accordingly, if there is no COP movement outside the stability zone, the score is 100%. If the COP moves outside the stability zone, the score decreases proportionally. The score reflects how much COP deviates from the center of the base of support based on population data [24].

Standing balance assessed with COP velocity—COP velocity while standing is a valid test of standing balance [25,26]. In this version of this test, patients were instructed to minimize the movement of a purple dot on the monitor, which represented the movement of their body’s COP. Patients were instructed try to minimize the movement of their COP represented by the purple dot on the monitor thus receiving continuous visual feedback. Three trials were conducted for 30 s each, and the single score with the average velocity of sway (cm·s^−1^) were used in the analyses [27].

Pain severity—CGH usually starts as intermittent pain and may progress to continuous pain originating at the back of the neck, especially upper cervical segments (C1–C3) [28]. We measured neck pain by a visual analog scale (VAS), consisting of a 100 mm horizontal line with the description “no pain” on the far left and “worst possible pain” on the far right. Patients were asked to rate their neck pain by placing a mark on the line corresponding to their current perceived level of pain. The distance along the line from the “no pain” marker was measured with a ruler giving a pain score out of 10 [29].

### 2.4. Statistical Analyses

The variables were normally distributed based on the Shapiro–Wilk test, and the between-group variances were equal based on the Levene’s test. Unpaired *t*-test was used to determine if the groups differed in age, height, mass, and BMI. Group by time analysis of variance with repeated measures on time was used to determine the effects of interventions on the outcomes. Time main effects and group by time interaction effects were characterized by partial eta squared (η_p_^2^) effect size. Cutoffs for η_p_^2^ are ≥0.01 (small), ≥0.06 (medium), and ≥0.14 (large). In case of a significant interaction, Tukey’s post hoc contrast was used to determine the means that differed at *p* < 0.05. Because the group sample sizes were relatively small, instead of Cohen’s d, the within-group changes over time were further characterized by Hedge’s g effect sizes. Cutoffs for g are ≥0.20 (small), ≥0.50 (medium), and ≥0.80 (large). We conducted the correlation analysis using the correlation coefficient (Pearson’s r) to quantify the strength and direction of the relationship between pairs of variables. The level of significance was set at *p* < 0.05. Analyses were conducted using IBM SPSS Statistics for MacIntosh, Version 29.0 (IBM Corp. Released 2023., Armonk, NY, USA, IBM Corp.).

## 3. Results

There was no significance difference between the control (CON) and the experimental (EXP) groups in sex distribution, age, body mass, height, and body mass index (BMI) (Table 1).

Intervention effects on sensory motor tests of postural control—In the sensory motor tests of postural control of the HSEO condition, the two interventions combined ((GDRE) plus selected physical therapy rehabilitation in the EXP) improved postural control by 8% (±7.35, time main effect: F = 40.9, *p* < 0.001, η_p_^2^ = 0.561) without a group by time interaction (*p* = 0.088).

In the HSEC condition, the two interventions combined improved postural control by 6% (±2.82, time main effect: F = 160.0, *p* = 0.001, η_p_^2^ = 0.833) without a group by time interaction (*p* = 0.126).

In the SSEO condition, the two interventions combined improved postural control by 5% (±4.51, time main effect: F = 46.2, *p* = 0.001, η_p_^2^ = 0.591) without a group by time interaction (*p* = 0.308).

In the SSEC condition, the two interventions combined improved postural control by 13% (±6.64, F = 186.3, *p* = 0.001, η_p_^2^ = 0.853) with a group by time interaction (F = 13.0, *p* = 0.001, η_p_^2^ = 0.289). Post hoc analysis showed that the 16% (±6.62, g = 2.6) improvement in EXP was greater than the 9% (±4.74, g = 2.1) improvement in CON.

Intervention effects on COP velocity during standing—The two interventions combined improved sway velocity by 9% (±11.90, F = 27.3, *p* = 0.001, η_p_^2^ = 0.460) with a group by time interaction (F = 20.9, *p* = 0.001, η_p_^2^ = 0.395). Post hoc analyses showed that the 17% (±11.79, *p* < 0.001, g = 0.9) improvement in EXP was greater than the 1% (±4.41, *p* > 0.05, g = 0.1) improvement in CON.

**Figure 2:**
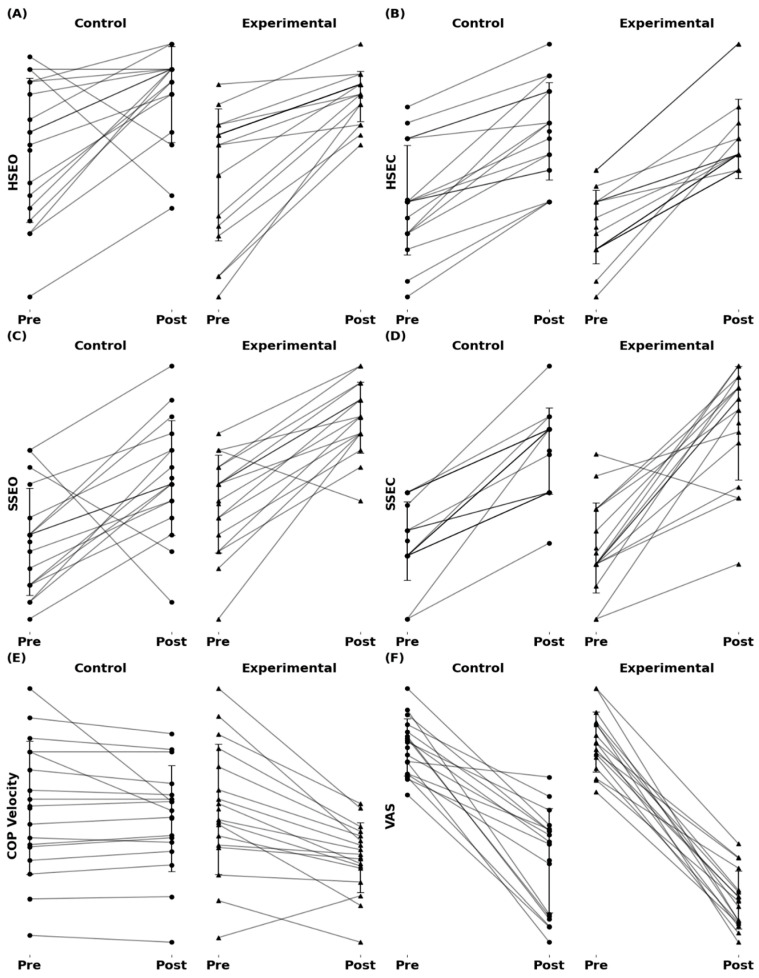
Individual pre- and post-intervention data for six outcomes. Each symbol represents one patient. Intervention effects are shown for: (**A**) HSEO (hard surface, eyes open), (**B**) HSEC (hard surface, eyes closed), (**C**) SSEO (soft surface, eyes open), (**D**) SSEC (soft surface, eyes closed), (**E**) COP (center of pressure velocity, cm·s^−1^), and (**F**) VAS (visual analog scale of neck pain, mm). Units for (**A**–**D**) are % relative to population data. Pre = before intervention, Post = after intervention.

Intervention effects on neck pain—The two interventions combined improved neck pain by 38 mm (±15.04) or 59% (±20.35) (F = 270.5, *p* < 0.001, η_p_^2^ = 0.894) with a group by time interaction (F = 8.8, *p* < 0.006, η_p_^2^ = 0.215). The 45 mm (±11.32) or 69% (±12.75, g = 5.3) improvement was greater in EXP than the changes in CON (31 mm ± 15.44 or 50% ± 22.44, g = 2.4) (Table 2 and Table 3) (Figure 2).

**Figure 3:**
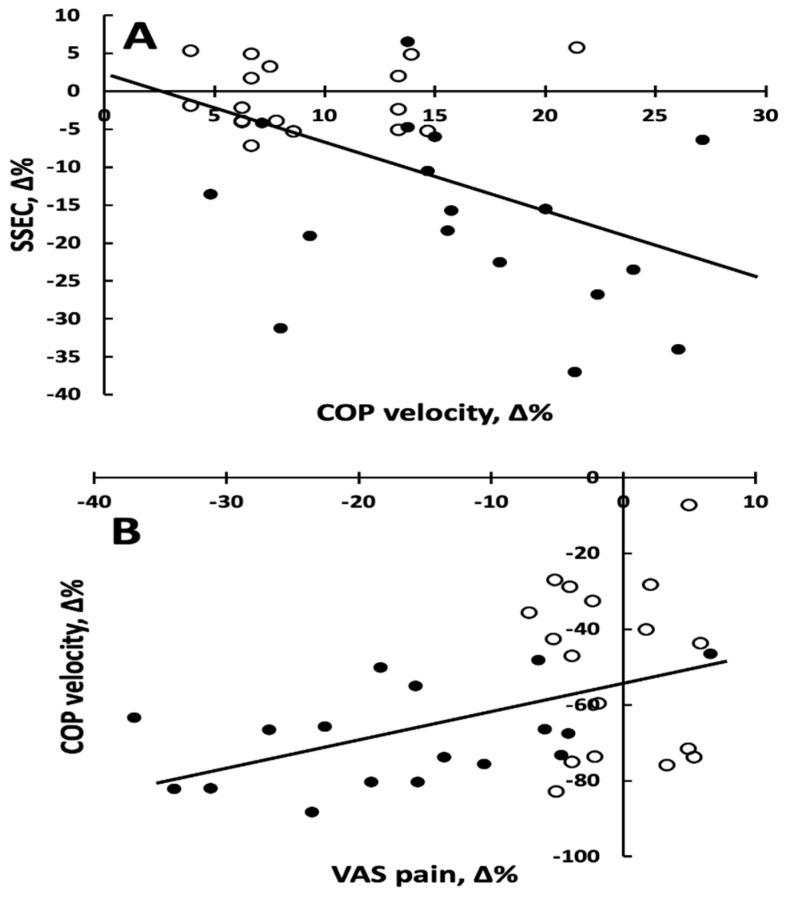
(**A**) Percent changes in SSEC (soft surface, eyes closed) versus percent changes in COP velocity. (**B**) Percent changes in COP velocity versus percent changes in neck pain. In both panels, open symbols (n = 17) represent the control group, and filled symbols (n = 17) represent the ex-perimental group.

Correlation analyses—Of those variables that showed a group by time interaction, the percent changes in condition SSEC vs. percent changes in COP velocity (Figure 3A) and percent changes in COP velocity vs. percent change in neck pain (Figure 3B) correlated moderately but significantly.

The relationship between percent change (∆%) in sensory motor measure of standardized postural sway while standing on a foam with eyes closed (SSEC) and percent change in center of pressure velocity (COP) while standing on a hard surface with eyes open. The relationship is characterized by the equation y = −0.87x + 2.5, r = 0.48, and *p* = 0.004 as shown in Figure 3A.

The relationship between percent change (∆%) in center of pressure velocity (COP) while standing on a hard surface with eyes open and the percent change in neck pain measured on VAS. The relationship is characterized by the equation y = 0.75x − 52.5, r = 0.44, and *p* = 0.008 as shown in Figure 3B.

## 4. Discussion

We examined the effects of a specific form of proprioceptive neck training on postural stability and neck pain in CGH patients. We found that improvements were greater in the experimental vs. the control group in the most challenging sensorimotor postural test (standing on foam with eyes closed), standing sway velocity without feedback, and neck pain. We discuss these findings with a perspective on managing and rehabilitating individuals with CGH.

We observed a time main effect in each of the six variables analyzed. These data suggest that a strong and consistent treatment effect for the two interventions combined (Figure 2, Table 2). However, we also observed strong group by time interaction effects in favor of the EXP vs. CON group, implying that the EXP group may have experienced more pronounced benefits from the proprioceptive training intervention. Indeed, the interaction effects occurred in the most difficult mCTSIB test when patients stood with eyes closed on an unstable surface, i.e., foam, so that EXP vs. CON improved, respectively, 16% and 9% (Figure 2D, Table 2). This test challenges the proprioception strongly [30]. These findings are consistent with previous studies highlighting the role of proprioceptive training in enhancing balance control in various patient populations [19,31].

Similarly, EXP (17%) vs. CON (1%) experienced significantly greater improvements in sway velocity (COP velocity) following proprioceptive training, indicating improved standing balance in CGH patients [32]. Postural stability depends on proprioceptive information from mechanoreceptors and vestibular and visual input to the central nervous system [33]. The moderate but significant association between the improvements in postural control under sensory challenge (SSEC) and COP velocity of sway (Figure 3) points to common elements in the two tests that were favorably influenced by the proprioceptive training.

Perhaps the most intriguing and clinically relevant finding is the 45 mm or 69% (EXP) vs. 31 mm or 50% improvement (CON) in neck pain (Figure 2F, Table 2). Headaches can arise from neck pain or neck muscle fatigue because such signals can interfere with sensory input from neck proprioceptors [8,12]. This type of interference can in turn affect postural control [19]. Neck proprioception, pain, and postural control are closely linked in both healthy individuals and those suffering from chronic neck pain [13]. Our data reveal that the GDRE was effective to preferentially reduce neck pain in EXP possibly by acting on cervical proprioception and neck muscle coordination, alleviating pain in cervicogenic headache [14,15]. The moderate but significant association between improvements in COP velocity and reductions in pain is perhaps one of the first indications to lend support for the proprioception-postural control-pain triangle (Figure 3).

The favorable effects of GDRE intervention on pain also point to the importance of engaging patients in active treatment vs. the traditionally used passive modalities, with a special emphasis on proprioception [34,35]. Indeed, three weeks of proprioceptive training vs. standard care improved significantly more chronic neck pain patients’ joint position sense, neck pain, and neck disability perception [36]. Further studies are still needed, as a review stated that there is still insufficient evidence if adding proprioceptive training to standard care in patients with chronic neck pain would reduce neck pain [37]. In total, the present data suggest that incorporating balance training through proprioceptive exercises targeting the cervical region, including GDRE, can provide evidence-based reductions in neck pain in CGH patients.

Several possible mechanisms may underlie the improvements in postural control in CGH patients. Cervical muscle spindles are important proprioceptors in maintaining postural stability [38]. The enhancement of suboccipital muscles in CGH patients is responsible for accurate kinesthesia and proprioception. These muscles modulate postural reflexes, crucial for eye–head coordination and postural stability. The suboccipital muscles are part of the same superficial back line of myofascial chains as the hamstring and calf muscles; all these muscles are involved in maintaining postural stability [36,39,40,41,42,43].

Apart from the observed sensory-motor changes, the GDRE intervention may have enhanced neuromuscular adaptation, which is essential in the management of CGH patients. It is likely that proprioceptive training improved neuromuscular communication through muscle-spindle integration and the central nervous system [38]. As a result, this helps improve cervical spine maintenance through enhanced postural control and muscle coordination. In addition, it has been established that proprioceptive training improves sensory input integration, which assists in reducing compensatory strategies that would otherwise aggravate the symptoms in CGH patients. Changes in postural control do not result solely from an enhancement in balance but also from the performance of more skilled movements in the muscle coordination of a person during challenging tasks.

This study also brings the wider significance of proprioceptive training for other patient groups suffering from chronic pain and balance disorder. It is likely that other patients who face proprioceptive deficits due to a variety of musculoskeletal conditions such as instability may gain benefits from similar training protocols. More studies should conduct investigations in order to determine whether patients with other forms of chronic headache, or balance dysfunction, are equally capable of achieving similar results in terms of the postural stability index, pain levels, and self-reported functional measures. Furthermore, these studies may address the issue of the duration of the effects of proprioceptive training in the context of postural control improvement and pain reduction.

## 5. Limitations and Conclusions

One limitation is the relatively small sample size that prevented us from examining any effects related to sex. However, we did observe several group by time interactions, suggesting that the study was powered reasonably. Another limitation is a lack of follow-up; thus, we cannot tell how long the preferential effects of GDRE lasted after the treatments were stopped. This is important as exercise effects often diminish once the intervention is stopped. Given these limitations, it is essential to emphasize that this work is a pilot study. Future research with larger sample sizes and follow-up periods is still needed to validate our findings and explore the effects of GDRE more thoroughly.

We did not measure cognition, behavior, or work performance. A lack of assessor blinding to group allocation could have introduced bias in the post-test data. It is possible that the Hawthorne effect biased our results: EXP could have improved simply because it received extra treatment and was closely observed. Patients could have modified their physical activity, diet, and medication schedule during the study period affecting the results, but we did not measure these factors. Finally, we did not measure any blood markers, neck muscle activation, or brain imaging to probe the potential mechanisms underlying the preferential effects of GDRE on postural control and pain.

In conclusion, while we did not examine the underlying mechanisms, proprioceptive training in the form of GDRE can improve selected measures of postural stability, standing balance, and neck pain in cervicogenic headache patients.

## Figures and Tables

**Table 1:** Demographic data of participants in the control (CON) and experimental (EXP) groups. The table describes the uniform distribution of baseline characteristics (gender, age, height, weight, and BMI in male and female participants) among the two rehabilitation groups.

	CON, n = 17	EXP, n = 17
	Mean (±SD)	Mean (±SD)
Female, n	11	8
Male, n	6	9
Age, y	41.2 ± 4.47	39.4 ± 3.53
Mass, kg	72.4 ± 5.20	70.1 ± 6.23
Height, m	1.72 ± 0.05	1.70 ± 0.06
BMI, k·m^2^	24.5 ± 0.83	24.2 ± 0.72

Values are mean ± standard deviation or frequency, n.

**Table 2:** Test statistics for the group by time analysis of variance with repeated measures on time for six outcomes.

TEST	F	*p*	ηp^2^	Power
**HSEO**	3.098	0.08	0.008	0.40
**HSEC**	2.047	0.12	0.07	0.33
**SSEO**	1.071	0.30	0.03	0.17
**SSEC**	13.028	0.001	0.289	0.93
**COP**	20.890	0.001	0.395	0.99
**VAS**	8.75	0.006	0.215	0.81

F, Fisher’s F statistic, *p*, value of probability, ηp^2^ partial eta squared, HSEO (hard surface, eyes open), HSEC (hard surface, eyes closed), SSEO (soft surface, eyes open), SSEC (soft surface, eyes closed); and as center of pressure velocity (COP, cm·s^−1^) or visual analog scale (VAS, mm). Significant time effects are noted where *p* < 0.05.

**Table 3:** Within-group differences (time effect of the intervention over groups) summarize the intervention effects on balance and pain outcomes for control (CON) and experimental (EXP) groups.

		Baseline (T1)	Post-Treatment (T2)	Within-Group Difference T1 to T2		
		Mean (SD)	Mean (SD)	MD	g	*p*
**HSEO**	CON	83.58 ± 5.69	88.00 ± 3.81	−4.42	0.89	0.003
	EXP	85.06 ± 6.53	92.82 ± 2.48	−7.76	1.53	0.001
**HSEC**	CON	85.12 ± 3.46	89.47 ± 3.08	−4.35	1.29	0.001
	EXP	86.42 ± 2.32	92.00 ± 2.50	−5.58	2.25	0.001
**SSEO**	CON	84.58 ± 3.18	88.35 ± 3.41	−3.77	1.10	0.001
	EXP	85.82 ± 2.89	90.94 ± 2.11	−5.12	1.93	0.001
**SSEC**	CON	76.17 ± 3.11	83.29 ± 3.42	−7.12	2.1	0.001
	EXP	75.71 ± 3.73	87.94 ± 5.23	−12.23	2.6	0.001
**COP**	CON	79.17 ± 7.02	87.17 ± 3.11	−8	0.1	0.001
	EXP	81.17 ± 8.28	91.12 ± 5.44	−9.95	0.9	0.001
**VAS**	CON	62.5 ± 11.31	31.2 ± 13.70	34.3	2.4	0.001
	EXP	65.4 ± 8.53	20.4 ± 8.24	45	5.3	0.001

HSEO (hard surface, eyes open), HSEC (hard surface, eyes closed), SSEO (soft surface, eyes open), SSEC (soft surface, eyes closed); and as center of pressure velocity (COP, cm·s^−1^) or visual analog scale (VAS, mm). Significant effects are noted where *p* < 0.05, g, Cohen’s effect size for small sample sizes, MD, mean differences.

## Data Availability

The data presented in the study are available upon request from the corresponding author.
